# A mutation in the conserved NPxY motif of β integrin PAT-3 alters outgrowth of neuronal dendritic spines

**DOI:** 10.17912/micropub.biology.002399

**Published:** 2026-10-31

**Authors:** Michele L Lemons, Noelia Genao, Hailey McKillop, Michael M Francis

**Affiliations:** 1 Department of Biological and Physical Sciences, Assumption University, Worcester, MA, USA; 2 Department of Neurobiology, University of Massachusetts Chan Medical School, Worcester, MA, USA

## Abstract

Integrins are heterodimeric transmembrane receptors that are critical in many biological processes, including neuronal development. Here we investigate integrin signaling in the outgrowth of dendritic spines on *C. elegans* GABAergic neurons using mutations engineered within the membrane-distal NPxY motif of the β integrin subunit PAT-3 . We found a Y/E substitution, but not Y/A or Y/F substitutions, reduced dendritic spine number and misoriented spines. The Y/E substitution in the NPxY motif also altered the distribution of the presynaptic scaffold CLA-1 , suggesting roles in both pre- and postsynaptic development. Our findings implicate integrin activation through the NPxY motif in synapse formation/maturation.

**Figure 1. A Y/E substitution in the membrane distal NPxY motif of the cytoplasmic tail of β integrin/ pat-3 disrupts dendritic spines:** A) Maximum projection confocal image of the *C. elegans* GABAergic DD1 neuron dendritic shaft (inverted LUT grey scale) labeled by transcriptional expression of myristoylated GFP (myrGFP). *pGABA* ::myrGFP in top image refers to cell specific transcriptional expression of myrGFP in DD GABAergic neurons using the *flp-13* promoter. Bottom panel is a cartoon representation of the top image and includes red carets pointing to ventrally directed spines protruding from the dendritic shaft. All images are from larval stage 4 animals and oriented with anterior to the left and ventral towards the bottom, as indicated by arrows. Scale bar, 5 μm. B) Inverted maximal projection images showing DD1 dendritic spines of wildtype, *pat-3 ( kq8041 )* , *pat-3 ( kq8042 )* and *pat-3 ( kq8043 )* animals. Scale bar, 5 μm. C) Scatterplot of the total number of spines within a 15 μm region of DD1 neurons in wild-type animals and *pat-3* mutants. The number of spines in *pat-3 ( kq8042 )* animals is significantly decreased compared to wildtype, *pat-3 ( kq8041 )* , and *pat-3 ( kq8043 )* animals. Each data point represents a measurement from an independent animal. Red horizontal bar indicates the mean. *p<0.05, **p<0.005, *** p<0.0005, Kruskal Wallis test. D) Number of dorsally directed spines per animal shown as a scatterplot. Dorsally directed spines are significantly increased in *pat-3 ( kq8042* ) compared to wild type. Red horizontal bar indicates the mean. Inset image of *pat-3 ( kq8042 )* animal with carets pointing to dorsally directed spines. *p<0.05, **p<0.005, ***p<0.0005, Kruskal Wallis test. Scale bar, 2 μm. E) Maximum projection confocal images of *C. elegans* GABAergic DD1 neuron dendritic shaft and spines (red) and presynaptic protein CLA-1 (green). *pGABA* ::mCherry refers to transgenic expression of the red fluorescent marker mCherry in GABAergic DD neurons ( *flp-13* promoter). *pACh* :: CLA-1 ::GFP refers to expression of the active zone protein CLA-1 tagged with green fluorescent protein (GFP) in cholinergic neurons ( *unc-17* beta promoter), including those that are presynaptic to GABAergic DD motor neurons. Representative images of wild type (left) and *pat-3 ( kq8042 )* mutants (right) are shown. Scale bar, 5 μm. Scatterplots showing the average number (F) and size (G) of CLA-1 ::GFP puncta per animal within a 50 μm anterior most portion of ventral nerve cord. Each dot represents a single animal. The number of CLA-1 ::GFP clusters is decreased and the size of CLA-1 ::GFP clusters is increased in *pat-3 ( kq8042* ) animals compared to wild-type controls. ***p<0.0005, **p<0.005, Mann Whitney test.

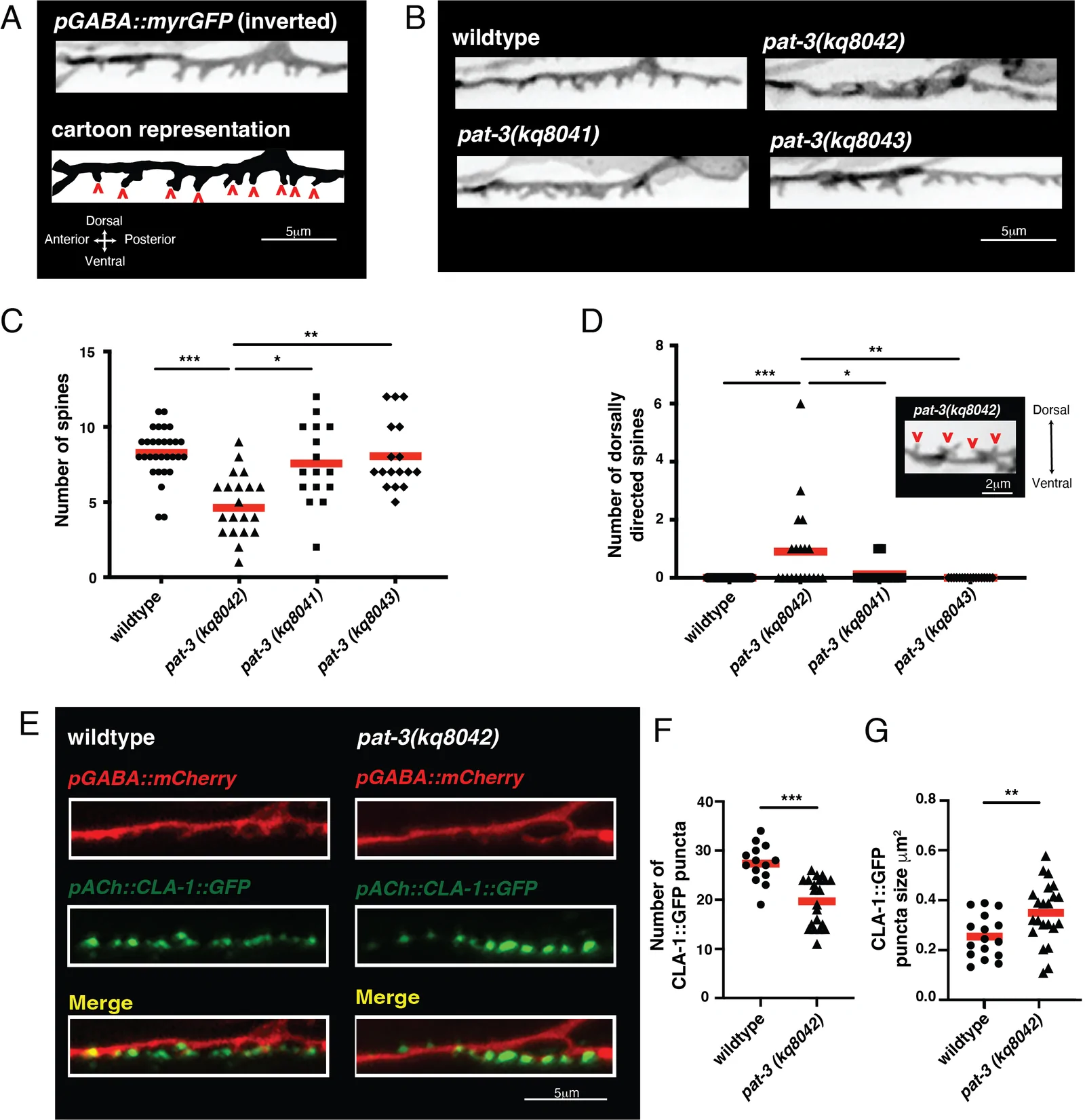



## Description

Synapse formation involves a sequence of carefully orchestrated molecular interactions to concentrate synaptic vesicles and release machinery at precisely defined sites opposite clusters of post-synaptic neurotransmitter receptors (Drapeau et al., 1995; Waites et al., 2005). Many studies have identified shared groups of proteins that form the core pre- and post-synaptic scaffolds, but the events that initiate synapse assembly and maturation often vary at the level of individual neurons and synapses. Mounting evidence indicates that integrin family members have key roles in synapse assembly and maturation (de Wit & Ghosh, 2016; Dityatev et al., 2010; Lilja & Ivaska, 2018; Park & Goda, 2016). Integrins are transmembrane extracellular matrix receptors that function as αβ heterodimers (Campbell & Humphries, 2011; Giancotti & Ruoslahti, 1999; Hynes, 1992, 2002). Integrins bind a variety of ligands including extracellular matrix, cell adhesion and axon guidance proteins, as well as intracellular signaling machinery such as talin and kindlin (Humphries et al., 2006; Moser et al., 2009; Nakamoto et al., 2004; Sonnenberg, 1993; Ye et al., 2014). At least 24 different integrin combinations are formed from 18 α and 8 β subunits in mammals, while the invertebrate model *C. elegans* forms only two integrin heterodimers (Fassler & Meyer, 1995) composed of one α subunit ( INA-1 or PAT-2 ) and a sole β subunit ( PAT-3 )(Gettner et al., 1995; Kramer, 2005).

Integrin function is greatly influenced by conformational state, integrins shift between low- (inactivated) and high-ligand affinity (activated) states. Integrin activation can be triggered by cytoplasmic signaling, known as “inside-out” activation (Calderwood, 2004a; Ginsberg, 2014; Kim et al., 2011; Liddington & Ginsberg, 2002; Wang, 2012). Inside-out activation is mediated through interactions between phosphotyrosine binding (PTB) domain proteins (including talin, kindlin) and cytoplasmic NPxY (asp-pro-x-tyr) motifs in the β integrin cytoplasmic domain (Bumm et al., 2025; Czuchra et al., 2006; Hanna et al., 2020; Jannuzi et al., 2002; Jannuzi et al., 2004; Kaapa et al., 1999; Lee et al., 2001; Sakai et al., 1998). The cytoplasmic tail of β integrin has two NPxY motifs, the membrane-proximal motif is proposed to interact with talin while the membrane-distal motif has been shown to interact with kindlin (Morse et al., 2014). Tyrosine phosphorylation of the NPxY motifs regulates the binding of PTB domain containing proteins, implicating NPxY motifs as molecular switches for integrin activation and signaling (Bledzka et al., 2010; Calderwood, 2004a, 2004b; Calderwood et al., 2013; Calderwood et al., 2002; Liddington & Ginsberg, 2002). While null mutations in the gene encoding the *C. elegans* β integrin *pat-3* are lethal, viable CRISPR-engineered mutations targeting the membrane-distal NPXY motif of PAT-3 were recently generated (Bumm et al., 2025; Hanna et al., 2020). Building upon previous studies from our lab and others that implicate integrins in the outgrowth of GABAergic axons (Baum & Garriga, 1997; Chisholm et al., 2016; Forrester & Garriga, 1997; Oliver et al., 2019; Poinat et al., 2002), we sought to employ these NPxY engineered mutants to investigate integrin signaling in the formation of previously characterized dendritic spines in *C. elegans* GABAergic neurons (Cuentas-Condori et al., 2019; Lemons et al., 2024; Oliver et al., 2022; Oliver et al., 2026; Philbrook et al., 2018).

To investigate the role of integrin activation through the β integrin/ PAT-3 NPxY motif in spine outgrowth, we used cell-specific expression of myristoylated green fluorescent protein (myrGFP) to visualize GABAergic dendrites in 3 available strains carrying CRISPR-engineered mutations in the conserved tyrosine residue of the PAT-3 membrane distal NPxY motif: wild type ^801^ NPVY ^804^ to NPVA ^804^ [alanine, *pat-3 ( kq8041 )* ], NPVE ^804^ [glutamate, *pat-3 ( kq8042* )], or NPVF ^804^ [phenylalanine, *( pat-3 ( kq8043 )* ] (Bumm et al., 2025; Hanna et al., 2020). These mutations are expected to disrupt the tyrosine residue (A), mimic tyrosine phosphorylation (E), or mimic non-phosphorylatable tyrosine (F), respectively. Consistent with previous studies (Lemons et al., 2024; Oliver et al., 2022; Philbrook et al., 2018), we observed roughly 8 dendritic spines in a 15 μm region anterior to the DD1 GABAergic neuron cell body of wild-type larval stage 4 (L4) animals ( Figure 1A- C). The number of dendritic spines on GABAergic DD1 neurons was significantly reduced by 43±6% in *pat-3 ( kq8042 )* animals carrying a Y>E substitution ( Figure 1B, C). Surprisingly, some spines appeared dorsally rather than ventrally oriented in these animals ( Figure 1B, D), suggesting either misdirected spine growth or the possibility of ectopic synaptic contacts. Substitution of the negatively charged glutamic acid (E) for tyrosine (Y) in *pat-3 ( kq8042 )* animals is predicted to mimic tyrosine phosphorylation (Anthis et al., 2009; Qiu et al., 2019; Reszka et al., 1992). Previous studies suggest phosphorylation of the distal NPxY motif disrupts kindlin binding, therefore disabling kindlin's ability to co-activate integrin (Bledzka et al., 2010; Harburger et al., 2009). Our findings suggest that constitutive tyrosine phosphorylation disrupts dendritic spines, perhaps by impacting interactions with kindlin/ UNC-112 or other modulators of integrin activation that act through this motif.

To ask whether the effects of the PAT-3 Y/E substitution were specific to the post-synapse or affected both pre- and postsynaptic specializations, we examined the distribution of the GFP-tagged active zone marker CLA-1 (Xuan et al., 2017) in cholinergic neurons, which are presynaptic partners to GABAergic motor neurons. As expected, CLA-1 ::GFP puncta were observed on the ventral side of ventrally directed spines in wild-type controls ( Figure 1E ), indicating presynaptic specializations opposing the dendritic spines. We found that the number of CLA-1 ::GFP puncta within a 50 μm region of the anterior ventral nerve cord was significantly decreased in *pat-3 ( kq8042 )* animals compared to controls ( Figure 1F ), while the size of CLA-1 ::GFP puncta slightly increased ( Figure 1G ). These findings demonstrate that both pre- and postsynaptic specializations are disrupted in *pat-3 ( kq8042 )* animals.

We next examined dendritic spines in *pat-3 ( kq8041 )* and *pat-3 ( kq8043 )* animals. A tyrosine (Y) to phenylalanine substitution (F) in the NPxY motif in *pat-3 ( kq8043 )* animals is predicted to preserve hydrophobic interactions associated with tyrosine but eliminate phosphorylation, perhaps acting similarly to a non-phosphorylatable tyrosine residue (Chen et al., 2006; Xu et al., 2010). The tyrosine (Y) to alanine substitution (A) in the NPxY motif of *pat-3 ( kq8041 )* animals is predicted to disrupt interactions requiring the hydrophobic aromatic ring of tyrosine as well as phosphorylation at this site (Chen et al., 2006). Surprisingly, we found that spine number was not significantly different in either of these mutants compared to wild-type animals ( Figure 1B, C). Although work to date suggests the membrane-proximal and -distal NPxY motifs have independent binding partners, we can't rule out the possibility that redundancy across the motifs contributes to these findings.

Prior analysis of membrane distal NPxY substitutions revealed that all three substitutions produced defects in the migration of gonadal distal tip cells (Hanna et al., 2020). In contrast, our finding that solely the phosphomimetic Y/E substitution disrupts dendritic spines is more consistent with recent studies of neuronal function in these strains, where the phosphomimetic E substitution produced severe effects, while the A and F substitutions did not (Bumm et al., 2025). Together, our results argue that tyrosine phosphorylation at the distal NPxY motif of PAT-3 is not essential for spine outgrowth, but may instead have a modulatory role, perhaps by influencing integrin interaction with kindlin/ UNC-112 (Bledzka et al., 2010; Qadota et al., 2012).

## Methods

**Strains**

All strains used in these experiments are listed in Table 1. Strains are N2 Bristol strain derivatives (wildtype) and were maintained at room temperature on nematode growth media plates seeded with *E. coli* strain OP50 . Some strains were kindly shared by the Lee lab at Baylor University (Hanna et al., 2020). Transgenic strains were obtained by microinjection to achieve transformation (Mello et al., 1991) and identified by using co-injection markers. Integrated lines were produced with X-ray irradiation and outcrossed to wild type/ N2 Bristol eight times. Only larval stage 4 (L4) hermaphrodites were used in this study.

**Confocal microscopy and analysis**

Animals were immobilized in sodium azide (0.3 M) on a 2% agarose pad. Images of dendrites of the DD1 neuron shown in Panels A, B and D were obtained using a Yokogawa CSU-10 spinning disk confocal system on an upright Olympus BX51WI microscope equipped with a Hammamatsu C9100-13 EMCCD camera and 63X oil immersion objective. A Cobolt Calypso 491 nm laser was used for excitation, and images were acquired using Volocity 4.3 software (Perkin-Elmer). All images were obtained using identical imaging and laser settings. Z stack volumes were acquired using an objective-coupled Piezo. Image analysis was conducted using Fiji software (open source) as described previously (He et al., 2015; Lemons et al., 2024; Oliver et al., 2022; Philbrook et al., 2018). Briefly, spines that were at least 0.2 μm in length were counted within a 15 µm region of interest (ROI) anterior to the DD1 cell soma.

Images in Panel E were obtained on a Yokogawa CSU-Z1 spinning disk confocal mounted on a Nikon NiE upright motorized microscope equipped with a Nikon 60x oil immersion objective and a Prime BSI Express CMOS camera (Teledyne Vision Solutions). Z stack images were acquired using Nikon Elements software, using identical imaging and laser settings. CLA-1 ::GFP puncta number and size were measured within a 50 µm region of the anterior ventral nerve cord using the analyze particles function in Image J.

## Reagents

**Primers used to confirm *pat-3 ( kq8041 ), pat-3 ( kq8042 ) and pat-3 ( kq8043 ).***

Forward primer OMF 3153 (primer in Exon 7 *in pat-3* transcript): ggccacagataatgcaacag. Reverse primer OMF3141: (primer in downstream UTR): acaatttatcgctaaatactcgtt. PCR generated 436bp product. Product was sequenced using forward primer OMF3140 (in exon 7): catgatagatccgaatacgc

**Table 1. Worm strains**

**Table:** 

**Strain**	**Genotype**	**Description**	**Plasmid**	**Shortened name**
IZ3105	*ufIs175*	DD-specific myristoylated (myr) GFP transcriptional reporter (green)	*ufIs175 [P flp-13 :: myrGFP* + *P unc-122* ::GFP]	* pGABA::myrGFP = ufIs175*
BU8041	*pat-3 ( kq8041 ) III *	CRISPR engineered *pat-3* mutation changes NPVY to NPVA (Hanna et al., 2020)		
BU8042	*pat-3 ( kq8042 ) III *	CRISPR engineered *pat-3* mutation changes NPVY to NPVE (Hanna et al., 2020)		
BU8043	*pat-3 ( kq8043 ) III *	CRISPR engineered *pat-3* mutation changes NPVY to NPVF (Hanna et al., 2020)		
IZ4167	*pat-3 ( kq8042 ) III; ufIs175*	DD-specific myrGFP transcriptional reporter (green)	*ufIs175 [P flp-13 :: myrGFP* + *P unc-122* ::GFP]	* pGABA::myrGFP = ufIs175*
IZ4225	*pat-3 ( kq8042 ) III; ufIs175*	DD-specific myrGFP transcriptional reporter (green)	*ufIs175 [P flp-13 :: myrGFP* + *P unc-122* ::GFP]	* pGABA::myrGFP = ufIs175*
IZ4226	*pat-3 ( kq8043 ) III; ufIs175*	DD-specific myrGFP transcriptional reporter (green)	*ufIs175 [P flp-13 :: myrGFP* + *P unc-122* ::GFP]	* pGABA::myrGFP = ufIs175*
IZ3973	*ufIs128 ; ufEx1679*	DD-specific mCherry transcriptional reporter (red), extrachromosomal array with cholinergic promoter driving CLA-1 ::green fluorescent protein (GFP) expression	*ufIs128 [P flp-13 ::mCherry+ Plgc-11::mCherry]; ufEx1679 [P unc-17 β::GFPnovo2:: cla-1* + *Plgc-11::GFP]*	* pGABA::mCherry = ufIs128* * pACh:: CLA-1 ::GFP = ufEx1679*
IZ4598	*pat-3 ( kq8042 ) III; ufIs128 ; ufEx1679*	DD-specific mCherry transcriptional reporter (red), extrachromosomal array with cholinergic promoter driving CLA-1 ::green fluorescent protein (GFP) expression	*ufIs128 [P flp-13 ::mCherry+ Plgc-11::mCherry]; ufEx1679 [P unc-17 β::GFPnovo2:: cla-1* + *Plgc-11::GFP]*	* pGABA::mCherry = ufIs128* * pACh:: CLA-1 ::GFP = ufEx1679*

**Table:** 
